# The Effect of Acupuncture and Moxibustion on Heart Function in Heart Failure Patients: A Systematic Review and Meta-Analysis

**DOI:** 10.1155/2019/6074967

**Published:** 2019-10-20

**Authors:** Bingxue Liang, Cui Yan, Lu Zhang, Zhonqi Yang, Lingjun Wang, Shaoxiang Xian, Lu Lu

**Affiliations:** ^1^The First Affiliated Hospital, Guangzhou University of Chinese Medicine, Guangzhou 510407, China; ^2^Lingnan Medical Research Center, Guangzhou University of Chinese Medicine, Guangzhou 510407, China; ^3^Key Laboratory of Chronic Heart Failure, Guangzhou University of Chinese Medicine, Guangzhou 510407, China

## Abstract

**Background:**

Acupuncture and moxibustion (A&M) has been used for treating heart failure in China since the Han Dynasty. This ancient therapy can be applied to many diseases according to the WHO recommendations. Although there are many clinical reports on the treatment of heart failure by A&M, its effectiveness is still not fully demonstrated. We aimed to systematically review the related randomized controlled trial (RCT) studies and conduct a meta-analysis.

**Methods:**

The PubMed, MEDLINE, EMBASE, AMED, CENTRAL, CNKI, Wanfang, and Weipu databases were searched electronically until December 2018. The data were extracted, and the risk of bias was evaluated. Meta-analysis, subgroup analysis, and metaregression were performed. Heart function was the main outcome assessed. The details of the intervention were also investigated.

**Results:**

Thirty-two RCTs involving 2499 patients were included. Most studies had an unclear risk regarding blinding and allocation concealment. Compared with the traditional treatment group, the experimental group had a higher efficacy rate (odds ratio (OR) = 2.61, 95% confidence interval (95%CI): = [1.84; 3.72], *I*^2^ = 0%, *p* < 0.0001) and a significantly improved left ventricular ejection fraction (LVEF) (mean difference (MD) = 6.34, 95%CI = [4.11, 8.57], *I*^2^ = 93%, *p* < 0.0001), cardiac output (CO) (MD = 1.02, 95%CI = [0.65, 1.39], *I*^2^ = 94%, *p* < 0.0001), 6-minute walk test (6MWT) (MD = 43.6, 95%CI = [37.43, 49.77], *I*^2^ = 0%, *p* < 0.0001), and reduced brain-type natriuretic peptide (BNP) (MD = −227.99, 95%CI = [−337.30, −118.68], I^2^ = 96%, *p* < 0.0001). Adverse events were inadequately reported in most studies.

**Conclusions:**

A&M may be a promising intervention as an adjunctive therapy to medication for treating heart failure. However, the evidence was inconclusive. Further large and rigorously designed RCTs are needed for verification.

## 1. Background

Heart failure (HF) is a common cardiovascular clinical syndrome and is defined as structural or functional problems in the heart that lead to insufficient oxygen supply to tissues and organisms. Although angiotensin-converting enzyme inhibitors (ACEi), angiotensin receptor blockers (ARB), beta-blockers, and aldosterone antagonists have an abundance of evidence for preventing HF progression, and diuretics, vasodilators, antihypertensive drugs are widely used to relieve symptoms, approximately 60%∼80% HF patients have died in 5 years [1]. Thus, refocusing on complementary and alternative medicines, such as acupuncture and moxibustion (A&M), is required.

A&M is one of the significant therapeutic modalities of traditional Chinese medicine (TCM) and can be dated back to thousands of years ago when Chinese ancestors used stone needles and moxa leaves for treating disease. In the history of TCM, there are many reports about A&M for treating heart failure-related symptoms, such as chest distress, shortness of breath, fatigue, and edema [2]. A&M is based on a unique and ancient medical system. The untouchable therapeutic theory provides a barrier for the modern healthcare system, which excludes A&M. However, there is increasing clinical evidence suggesting that A&M should not be ignored during clinical decisions. In recent years, many case reports and clinical trials demonstrating the efficacy of A&M in treating HF have been conducted, and many experts have recommended patients for A&M treatment to reduce drug dosages or relieve adverse events [3, 4]. However, as an ancient alternative treatment, whether A&M plays a role in curing HF awaits further study. To verify the efficacy of A&M in improving heart function, systematic reviews or meta-analyses are needed. This study is designed to assess the effect of A&M therapy on HF and related factors that may influence the curative effect.

## 2. Methods

### 2.1. Protocol and Registration

The study is registered with PROSPERO, number CRD42018105038.

### 2.2. Eligibility Criteria

The included studies met the following eligibility criteria: (1) Studies were designed up to randomized controlled trial (RCT) standards. (2) Patients were diagnosed with either chronic heart failure (CHF) or acute heart failure (AHF). (3) No restriction was imposed on the cause of HF, apart from cases due to pregnancy, chemotherapy, congenital deficits, or surgery. (4) No restriction was imposed on gender, ethnicity, ejection fraction, systolic/diastolic HF, clinical setting, or left-/right-sided HF. (5) The experimental group was treated with manual acupuncture, electroacupuncture, auricular acupuncture, needle-warming acupuncture or moxibustion, and pharmaco-acupuncture, laser acupuncture, or other uncommon forms of acupuncture were excluded. (6) The control group was treated with placebo (sham) or an active control procedure, such as no treatment, sham acupuncture, or conventional medication treatments. (7) The studies that compared two acupuncture therapies directly were excluded.

### 2.3. Information Sources

Electronic searches were conducted at the following databases by two independent authors (LBX and YC) from inception until December 2018: MEDLINE, EMBASE, Cochrane Central Register of Controlled Trials (CENTRAL), China National Knowledge Infrastructure (CNKI), Wanfang, and Weipu databases. There were no language restrictions imposed in this study. To find unreported data, conference proceeding lists in major Chinese journals were manually searched, and corresponding authors were contacted via mail if further information was necessary.

### 2.4. Literature Search Strategy

Search strategy in Medline, for example:

1. acupuncture [Mesh]
2. acupuncture [tiab]
3. electroacupuncture [Mesh]
4. electroacupuncture [tiab]
5. “acupuncture therapy” [Mesh]
6. “acupuncture therapy” [tiab]
7. moxibustion [Mesh]
8. moxibustion [tiab]
9. “moxibustion therapy” [Mesh]
10. “moxibustion therapy” [tiab]
11. “heart failure” [Mesh]
12. “heart failure” [tiab]
13. “cardiac failure” [tiab]
14. “myocardial failure” [tiab]
15. “heart insufficiency” [tiab]
16. “cardiac insufficiency” [tiab]
17. “ventricular dysfunction” [Mesh]
18. “ventricular dysfunction” [tiab]
19. Or/1–10
20. Or/11–18
21. 19 and 20

### 2.5. Data Collection Process

Two independent researchers (LBX and YC) recorded the data from all the eligible articles in a predefined Excel format that included the following items: author, published year, country, participants, age, New York Heart Association (NYHA) level, course of disease, intervention, retention time, treatment duration and frequency, control types, and primary and secondary outcomes (i.e., heart rate (HR), 6-minute walk test (6MWT), left ventricular ejection fraction (LVEF), cardiac output (CO), stroke volume (SV), cardiac index (CI), brain natriuretic peptide (BNP), and high-sensitivity C-reactive protein (hs-CRP)). Disagreements were resolved by a third researcher (ZL). The primary author of the study would be contacted by e-mail if the data were unclear.

### 2.6. Data Items

The efficacy rate was the primary outcome, and other observed values that reflect cardiac function were HR, LVEF, CI, SV, CO, BNP, and 6MWT. The normal range of HR is 60–100 times/min; the standard range of LVEF is 55–80%; the normal values of CI, SV, and CO are 2.4–4.21/min/m^2^, 65–70 ml/time, and 5–6 L/min, respectively. NYHA grading was used to classify the impairment of cardiac function into four grades according to the degree of activity inducing the symptoms of HF. The scheme was proposed in 1928 and is still currently used because of its simplicity. LVEF is the percentage of stroke output to the volume of end-diastolic ventricle. LVEF is related to the contractility of the myocardium. CI is calculated by dividing the volume of blood pumped by the heart (L/min) by the surface area of the body (M^2^), so that patients of different body sizes can be directly compared. The 6MWT is mainly used to evaluate the efficacy of intervention in patients with HF, and cardiac function can be reflected and divided into 4 levels accordingly. SV refers to the amount of blood discharged by a single ventricle in a heartbeat, which is related to cardiac contractility, blood volume, and blood pressure. CO is the amount of blood that the ventricle pumps out per minute, which is related to SV and HR. As a quantitative marker of HF, BNP not only reflects left ventricular systolic dysfunction but also left ventricular diastolic dysfunction and right ventricular dysfunction. A BNP exceeding 400 pg/ml indicates that the patient is 95% likely to have HF. The 6MWT is mainly used to evaluate the efficacy of intervention in patients with HF, and cardiac function can be reflected and divided into 4 levels accordingly.

### 2.7. Risk of Bias in Individual Studies

The risk of bias (ROB) was assessed by two independent authors (LBX and YC) according to the Cochrane Handbook for Systematic Reviews. In the event of disagreement, a third reviewer (ZL) resolved the disagreement. ROB was assessed in the following domains: (1) random sequence generation, (2) allocation concealment, (3) blinding of participants and personnel, (4) blinding of outcome assessment, (5) incomplete outcome data, (6) selective reporting, and (7) other bias. In consideration of the features of A&M clinical research, other biases included the specifics of the operation. ROB was graded as “high” risk, “low” risk, or “unclear” risk by the Cochrane assessment tool.

### 2.8. Statistical Analysis

To pool the mean differences for HR, LVEF, CI, SV, CO, BNP, and 6MWT, we calculated the standardized mean difference (SMD) using Mantel–Haenszel's method to accurately approximate bias. To pool the associated risk, we used a generic method for meta-analyses based on inverse variance weighting according to Mantel–Haenszel's method. The heterogeneity of the effect size was evaluated using tau-squared statistics. A fixed-effect model was used when the tau-squared value was <50%; otherwise, a random effects model was used. We used funnel plots to investigate the publication bias in our meta-analysis. To explore the sources of heterogeneity, we performed a metaregression analysis with weight dependence.

All statistical analyses were performed using *R* statistical software (version 3.4.0; meta and metafor packages).

## 3. Results

### 3.1. Study Selection

**Figure 1:**
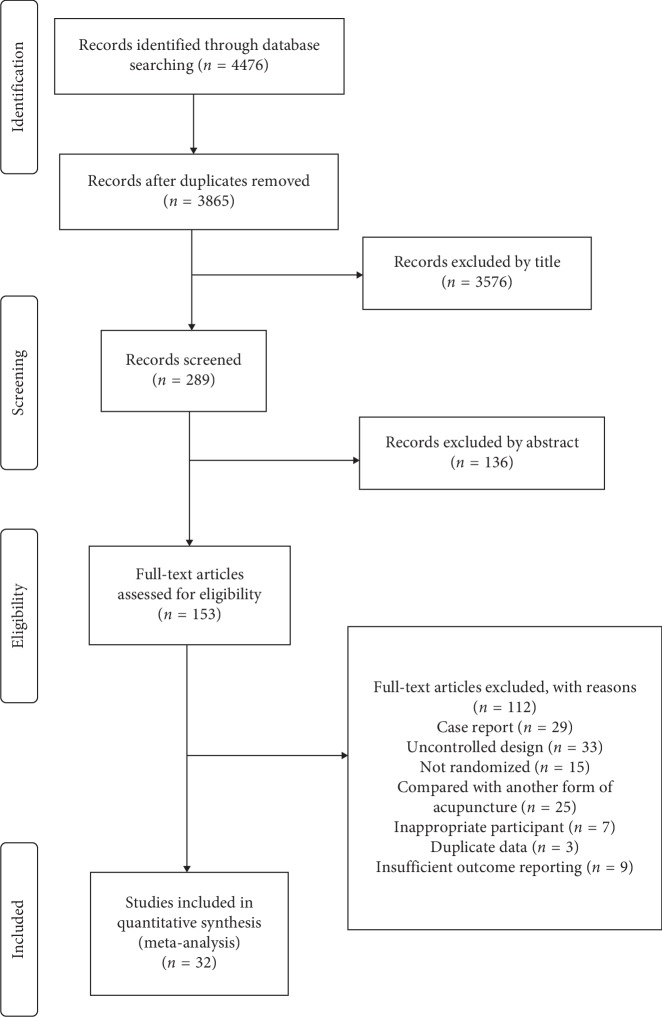
Study flow diagram.

The flow diagram of the study selection and identification is shown in Figure 1. A total of 4476 articles were identified by electronic and manual searching. After 611 duplicates were omitted, 3865 remained. Among these articles, 153 studies remained after screening the titles and abstracts. The full-length texts of the remaining articles were carefully reviewed, of which 112 studies were excluded. Finally, 32 RCT studies were included in the quantitative analysis [3, 5–35]. The characteristics of these 32 studies are shown in Table 1.

### 3.2. Study Characteristics

Thirty-two RCTs met the inclusion criteria: one study was conducted in Germany, one in the United States, and the other 30 studies in China. Among the studies, 4 focused on AHF and 28 focused on chronic heart failure. Table 1 shows the characteristics of the included studies. In total, 2499 patients (1308 in the acupuncture or moxibustion group, 1191 in the control group) participated. The number of participants ranged from 9 to 114 in the study groups (mean ± s.d., 40.875 ± 23.09) and 8 to 103 in the control groups (mean ± s.d., 37.219 ± 19.75). All studies included both men and women. The mean age of the participants was 62.974 ± 23.02 years. The NYHA protocol for heart function evaluation was used. Only two trials included NYHA grade I patients. Most of the trials included grade II–IV patients.

### 3.3. Findings of Included Studies

Eight trials observed the curative effect of moxibustion, and 24 trials observed acupuncture. There were 6 types of intervention and control methods (Table 2): (1) acupuncture with conventional therapy (CT) vs. sham acupuncture with CT; (2) acupuncture with CT vs. CT alone; (3) acupuncture plus TCM with CT vs. CT alone; (4) acupuncture plus TCM with CT vs. TCM with CT; (5) moxibustion with CT vs. CT alone; and (6) moxibustion plus TCM with CT vs. TCM with CT. The acupuncture therapy retention times ranged from 15 min to 60 min (mean ± s.d., 24.5 ± 10.4 min), and the treatment times ranged from 1 to 56 (mean ± s.d., 17.5 ± 17.2 min). Moxibustion therapy retention times ranged from 20 min to 240 min (mean ± s.d., 63.6 ± 79.1 min), and the treatment times ranged from 3 to 56 (mean ± s.d., 23.3 ± 18.4).

### 3.4. Risk of Bias Assessment

The overall ROB was high based on the Cochrane criteria. Particularly, the biases related to participant blinding and allocation concealment were not clearly described. The details of ROB are shown in Figures [Supplementary-material supplementary-material-1] and [Supplementary-material supplementary-material-1].

### 3.5. Evaluation of Heterogeneity and Meta-Analysis

Table 3 shows that the efficacy of A&M in the treatment of HF was 2.61 times higher than that in the control group (*p* < 0.0001), which can be reflected in the following aspects. In terms of vital signs, HR decreased by 2.63 times/min (*p* < 0.0001). In terms of cardiac ultrasonography, the average LVEF was increased by 6.34% (*p* < 0.0001), and cardiac volume per stroke (SV) was increased by 12.41 ml (*p* < 0.0001). In terms of biochemical indexes, BNP decreased by 227.99 mol/ml on average (*p* < 0.0001), *N*-terminal prohormone of BNP (NT-proBNP) decreased by 553.05 mol/ml (*p* < 0.0001), and hsCRP decreased by 1.61 mg/dl (*p* < 0.01). In terms of functional status, the distance of the 6MWT increased by 43.6 meters (*p* < 0.0001). The forest maps of the main results are shown in Figures [Supplementary-material supplementary-material-1]–[Supplementary-material supplementary-material-1]. However, there was significant heterogeneity between the included RCTs for different study designs, and the funnel plots are shown in Figures [Supplementary-material supplementary-material-1]–[Supplementary-material supplementary-material-1], showing some publication bias related to BNP.

### 3.6. Meta-Regression

Table 4 shows that the HR was inversely proportional to the length of each treatment; thus, the HR decreased with increasing length of acupuncture or moxibustion. BNP was inversely proportional to the total number of treatments; thus, the BNP concentration decreased with increasing frequency of acupuncture. A trend can also be seen in Figures [Supplementary-material supplementary-material-1] and [Supplementary-material supplementary-material-1].

## 4. Discussion

A&M is one of the oldest treatments in the world and has been used for thousands of years. During its 2500 years of development, A&M has accumulated rich experience and proved that it can effectively treat a wide range of diseases and conditions. In 2003, the WHO listed a total of nearly 100 kinds of diseases recommended using A&M [36]; cardiac diseases were included in this study. In the past, meta-analyses have shown that acupuncture can improve arrhythmias and myocardial ischemia [37, 38]. A recent multicenter large-scale clinical randomized trial showed that adjuvant acupuncture treatment can significantly improve the symptoms of patients with stable angina pectoris [39]. According to a literature review and meta-analysis [40–42], acupuncture can also improve the cardiac function of patients with HF, and a single session of physical therapy and the total course of treatment may affect the efficacy. This study further explored the effect of acupuncture on various cardiac function indicators in patients with heart failure and found that acupuncture has improved effects on major heart function indicators such as LVEF, BNP, and 6MWT in patients with HF. Through subgroup analyses, it was found that the effect of acupuncture and moxibustion on heart function was almost the same, except for the CO value, where the acupuncture effect may be better than that of moxibustion. The metaregression found that HR and BNP values were correlated with the duration of treatment.

When we selected the literature, we did not treat A&M differently, as in many similar studies, because in the overall thinking of TCM, A&M are inseparable. The *Essential Questions in Yellow Emperor's Inner Canon*, an ancient medical book, recorded the following: “Therefore, the sages can integrate comprehensive methods to treat similar diseases and obtain specific therapeutic effects for proper conditions. The reason why similar diseases with different manifestations could be cured is that the sages can obtain complete information and catch the general principle [43].” However, due to the smoke roasting, complex operation, and potential scald hazards of moxibustion, acupuncture is gradually starting to replace moxibustion in China. Even in developed countries, such as Europe and the United States, people may have only heard of acupuncture, ignoring moxibustion. Indeed, both A&M arose in the Stone Age, which together constituted the main body of the original rudiment of the Chinese medicine system. In theory, A&M are two sides of the same coin, and in application, these treatments are supplementary to each other. Sun Simiao, a famous doctor in the Tang Dynasty, said, “Every disease is blocked by Qi and blood that cannot be propagated to Zangfu-meridians. Needles are used to guide it, and moxibustion is used to warm it.” Thus, A&M are indispensable. Moxibustion was used in less than one-third of the 32 references included in our meta-analysis. Through subgroup analyses, we found that both acupuncture and moxibustion can improve the cardiac function of patients with HF, with similar efficacy. Except for the improvement in the CO value, acupuncture showed a slight advantage over moxibustion; however, due to the limited number and quality of studies included, no conclusive conclusions can be drawn. In fact, both A&M are methods based on the theory of meridians and acupoints, which take different forms of physical stimulation to adapt to different body states. In other words, acupuncture is mostly used for syndromes of excess and heat, while moxibustion is mostly used for syndromes of deficiency and cold.

The mechanism of the efficacy of A&M has been the focus of international research. In 2007, the American Acupuncture Research Association highlighted some problems that were difficult to solve in A&M research, such as the lack of significant difference between the experimental group and the placebo or sham A&M groups [44] and the significantly better effect of the sham A&M group than that of the placebo medicine group [45, 46]. The efficacy of placebo or sham A&M groups is related to the cognition, expectation, or attention of the patients to A&M therapy, the environment of the consulting room and the suggestions of the doctor. Since the professional operation of A&M, it is difficult to blind the operator, but patients can be blinded by false acupoints or false acupuncture, so it is difficult to avoid the placebo effect caused by the psychological hint or expectation of the doctor. Of the 32 studies included in this study, only 3 trials used placebo controls, and the efficacy of the experimental group was superior to that of the placebo control group. In fact, in China, where A&M originated, a sham acupuncture group was rarely set up as a placebo control in the study of clinical trials. Although such a trial design was considered unscientific by international researchers, it indirectly proved that Chinese researchers were not interested in whether A&M had specific efficacy beyond placebo. First, A&M is based on time-honored TCM theory and experience, and thousands of clinical experiences and efficacy demonstrations have proven the effectiveness of A&M. Second, the so-called placebo effect is strongly associated with psychological cues. However, A&M therapy has not rejected the role of psychological suggestions. A&M is also based on the theory of “keeping spirit,” “treating spirit,” and “regulating spirit.” Spirit is psychology, belief, concept, etc. Acupuncturists never reject the good effect of a psychological hint. Thus, psychological hint is one of the components of the curative effect of A&M.

Many animal experiments have explored the mechanism of acupuncture in the treatment of HF. Many scholars have conducted studies on acupuncture mediated by the sympathetic nervous system [42]. Many studies have shown that the acupuncture effect is closely related to the sympathetic nervous system, which is believed to play an extremely important role in the multipathway and multitarget acupuncture effects [47]. As shown in the study (Table 2), the improvement of A&M for patients with HF is comprehensive, and A&M can simultaneously intervene in multiple cardiac function-related indicators. However, the study also noted that a single acupuncture cannot reduce the resting sympathetic nerve activity of patients with HF, and the regulation of HR and blood pressure is not obvious [7]. Through a metaregression analysis, we found that the duration and number of treatments may affect the efficacy of A&M. By combing the included studies, we found that in most cases, the retention of acupuncture was 20–30 minutes, whereas that of moxibustion was approximately 40 minutes on average. Most studies were conducted over a course of 2 to 4 weeks, with sessions 3 to 7 times a week. Some studies have confirmed that needle retention is an important factor affecting the efficacy of acupuncture, which should be determined according to the disease, the constitution of the patient, the meridians and acupoints, and the length of the disease. Under certain stimulation intensities, the length of time of needling directly affects the curative effect of acupuncture. For example, stimulation of Neiguan can cause transient excitement of the vagus nerve in healthy people, and then the acupuncture effect gradually weakens and disappears. Needle retention can reduce HR for a longer time compared with nonretention [48].

In addition to inhibiting sympathetic activity, many animal experiments have explored other mechanisms of A&M in the treatment of HF and found that acupuncture can prevent fibrosis [49, 50], modulate inflammatory factors [51–53], inhibit the renin-angiotensin aldosterone system [54], improve the state of water sodium retention [55], reduce myocardial injury [56–59], protect myocardial contraction diastole function [60], inhibit myocardial hypertrophy, and reverse ventricular remodeling [50, 61].

Analysis of the acupoint groups included in the present study revealed that the most commonly used acupoints for the clinical treatment of HF were Neiguan, Shenmen, and Back-Shu points. Neiguan is located in the pericardium meridian of hand Jueyin, and its afferent neurons are mainly C5∼T1 spinal ganglion, converging with the cardiac afferent nerves at the C8∼T1 spinal cord. Shenmen is located in the Shaoyin heart meridian of the hand, and its afferent neurons are located in the T1∼T3 spinal ganglion, converging with the visceral afferent nerve that innervates the heart in the posterior horn of the upper thoracic medullary segment. Therefore, heart disease can be induced to the body surface of the medial area of the upper limb, and acupuncture of heart meridians can affect heart function. Back-Shu acupoints are very close to the dorsal root of spinal nerves, and the distribution law is roughly consistent with the segmental distribution characteristics of spinal nerves. Back-Shu acupoints can adjust somatic sensory nerve endings and sympathetic nerve endings and then act on the nerve center of the corresponding segment of the spinal cord to adjust the visceral function. Additionally, these acupoints can be transmitted to the brain by somatosensory fibers and visceral sensory fibers, realizing the benign adjustment of the whole body by the connection of downward conduction fibers related to the brain.

Although this study and many animal experiments as well as clinical trials have proven that A&M can improve the cardiac function of patients with HF to some extent, there are still many limitations in this study, mainly because the quality of the included clinical trials is not high. The blind method mostly adopts the random number table method, and some RCT trials do not explicitly record the random method; additionally, there is a lack of large multicenter stratified RCTs. In addition, only 4 trials had registered ethical supervision. The importance of ethics in clinical trials is not only limited to respecting the patients' right to know but also can play a supervisory role in regulating the operation of clinical trials. A total of 32 cases were included in the study, covering the period of 1993 to 2018. In 2005, the Chinese Medical Association began to pay attention to the issue of medical ethics and require editorial departments to increase the requirements for medical research ethics in submission notes. After more than a decade of popularization, this regulation has been gradually adopted by Chinese clinical research institutes. To avoid research bias, we conducted another meta-analysis on the studies with ethical records and which described EF values. The results were as follows: MD = 7.18, 95% CI = [4.04, 10.32], *I*^2^ = 69%, *p* < 0.0001, which were consistent with the conclusions obtained from the overall results.

Several implications of this review are presented for future research and practice. First, the single treatment time and the number of treatment times have different effects on the improvement of cardiac function. Follow-up clinical studies can set up treatment course groups to clarify the correlation. Second, many studies lack long-term follow-up. As a physical therapy with low side effects, acupuncture is suitable for long-term application, but no studies have observed the survival curve of acupuncture in the treatment of HF. Finally, A&M is a treasure left to the world by TCM, which has been included in the world cultural heritage list and is increasingly widely used. More standardized large-scale clinical trials are needed to further study the efficacy of A&M in the treatment of HF and contribute to the popularization of this therapy.

## 5. Conclusions

A&M can improve the cardiac function of patients with HF, and its mechanism, which has been confirmed by many clinical studies, is being explored continuously. However, the results are still inconclusive because of the limited quality and quantity of the included studies. Moreover, there is no evidence that acupuncture prolongs survival in patients with HF. Therefore, we look forward to high-quality, rigorous, large-scale, multicenter, randomized, controlled clinical studies that can minimize study bias and generate high-quality evidence.

## Figures and Tables

**Table 1:** Characteristics of the included studies.

Author	References	Country	Type of control (2)	Year	Age (1)	Disease	NYHA	No. of patients in the study group	No. of patients in the control group	Risk of bias (3)
Arnt V. Kristen	[3]	German	Acu plus med vs. placebo acu plus med	2013	60.3 ± 3.5	CHF	II-III	9	8	A
Qiusheng Xiao	[5]	China	Acu plus med vs. med	2014	67.3 ± 12.6	AHF	III-IV	30	30	C
Jiren Zhou	[6]	China	Acu plus med vs. med	1993	40.6 ± 2.9	CHF	III-IV	7	5	A
Holly R.	[7]	USA	Acu plus med vs. placebo acu plus med	2002	43 ± 11	AHF	II-III	10	20	A
Na Li	[8]	China	Mox plus TCM and med vs. TCM and med	2016	70 (60–80)	CHF	II-III	30	30	C
Jinling Zhao	[9]	China	Mox plus med vs. med	2018	66 ± 4	CHF	NA	35	35	C
Yanqin Sun	[10]	China	Mox plus med vs. med	2015	58 ± 17.6	CHF	II-III	60	60	B
Peng Deng	[11]	China	Mox plus med vs. med	2002	64 ± 9	CHF	III-IV	40	40	C
Jing Wang	[12]	China	Mox plus med vs. med	2012	66	CHF	II-IV	30	30	C
Yongjian Wen	[13]	China	Acu plus med vs. med	2012	67.21 ± 4.18	CHF	II-IV	67	67	C
Xin Li	[14]	China	Mox plus med vs. med	2013	84.68 ± 8.62	CHF	II-IV	35	35	C
Dongqun Lin	[15]	China	Acu plus med vs. med	2009	63.5 ± 7.2	CHF	II-IV	32	30	C
Xiaofeng Zheng	[16]	China	Acu plus med vs. med	2016	62.8 ± 5.4	AHF	III-IV	29	29	C
Weidong Huang	[17]	China	Acu plus med vs. med	1998	62.87	CHF	NA	114	9	B
Leiqun Cheng	[18]	China	Acu plus med vs. med	2017	59.17 ± 7.11	CHF	II–IV	50	50	C
Wei Gao	[19]	China	Acu plus med vs. med	2017	63 ± 6	CHF	I-II	40	40	C
Ziyong Li	[20]	China	Acu plus med vs. med	2012	65 ± 3	CHF	III-IV	22	18	C
Chunbai Lai	[21]	China	Acu plus med vs. med	2016	55 (40–70)	CHF	II–IV	42	43	C
Lin Wang	[22]	China	Acu plus TCM and med vs. med	2016	57.6 ± 11.3	CHF	NA	103	103	C
Yanli Rong	[23]	China	Mox plus med vs. med	2017	65.7 ± 8.6	CHF	I–IV	40	40	C
Lisha Mai	[24]	China	Mox plus med vs. med	2013	64.2 ± 2.3	CHF	II–IV	40	40	C
Qimei Zhang	[25]	China	Acu plus med vs. med	2008	61.5	CHF	II–IV	30	30	C
Zhaojia Chen	[26]	China	Acu plus med vs. med	2016	54.47 ± 1.55	CHF	II-IV	60	60	C
Jingjuan Yu	[27]	China	Acu plus med vs. med	2014	71 ± 6	CHF	II–IV	40	40	C
Dongmei Liu	[28]	China	Acu plus med vs. med	2015	60 ± 11	AHF	III-IV	28	21	C
Yongchang Ma [29]	[29]	China	Acu plus TCM and med vs. med	2016	65 ± 3	CHF	NA	40	40	C
Yanna Lei	[30]	China	Acu plus TCM and med vs. TCM and med	2010	72.5	CHF	II-IV	30	30	C
Xingliang Fan	[31]	China	Acu plus TCM and med vs. med	2016	68 ± 6	CHF	NA	34	32	C
Yong Zhi	[32]	China	Acu plus TCM and med vs. med	2016	52	CHF	II–IV	52	52	B
Chunying Si	[33]	China	Acu plus TCM and med vs. TCM and med	2014	73 ± 8	CHF	NA	16	16	C
Haifeng Zhou	[34]	China	Acu plus TCM and med vs. med	2009	46 (28–64)	CHF	II-III	60	55	C
Minyong Gan	[35]	China	Acu plus med vs. med	2018	60 ± 8	CHF	II–IV	53	53	C

(1) Age in years is presented as the mean ± SD or mean (range). (2) NA: not acquired; Acu: acupuncture; Mox: moxibustion; med: medicine; and TCM: traditional Chinese medicine. (3) Risk of bias: Grade A: low degree of bias, completely meets the quality standards of 4 or more items (low risk). Grade B: moderate bias, fully meets the quality standards of 2 or 3 items; Grade C: high bias, 1 item or more does not meet the standards completely.

**Table 2:** Details of the included studies.

Author	Outcomes	Intergroup difference	Study group	Control group	Mean difference
			Mean (SD)	Mean (SD)	MD [95% CI]
Arnt V. Kristen	LVEF	*p* > 0.05	2 (11.53)	1 (10.49)	1 [−9.47; 11.47]
	6MWT	*p* < 0.05	42 (154.05)	−5 (171)	47 [−118.66; 212.66]

Qiusheng Xiao	HR	*p* > 0.05	−21.1 (18.58)	−18.6 (19.44)	−2.5 [−12.12; 7.12]
	CI	*p* < 0.05	1.3 (0.46)	0.9 (0.43)	0.4 [0.17; 0.63]
	MAP	*p* > 0.05	−13.2 (16.11)	−12.5 (16.2)	−0.7 [−8.88; 7.48]
	SI	*p* < 0.05	18.2 (6.24)	13.5 (6.27)	4.7 [1.53; 7.87]

Jiren Zhou	CO	*p* < 0.01	0.82 (0.52)	−0.16 (0.47)	0.98 [0.42; 1.54]
	SV	*p* < 0.05	8.25 (5.09)	−3.68 (4.42)	11.93 [6.52; 17.34]
	CI	*p* < 0.01	0.46 (0.41)	−0.16 (0.36)	0.62 [0.18; 1.06]

Holly R.	HR	*p* > 0.05	0 (14.53)	1 (16.54)	−1 [−14.65; 12.65]
	MAP	*p* > 0.05	−1 (10.54)	2 (9.29)	−3 [−11.71; 5.71]

Na Li	BNP	*p* < 0.05	−1481.81 (459.14)	−1252.19 (397.87)	−229.62 [−447.02; −12.22]
	CRP	*p* < 0.05	−11.99 (1.29)	−9.59 (1.12)	−2.4 [−3.01; −1.79]
	6MWT	*p* < 0.05	157.28 (67)	133.57 (64.56)	23.71 [−9.59; 57.01]

Jinling Zhao	NT-proBNP	*p* < 0.01	−1892.24 (752.62)	−1406.51 (683.93)	−485.73 [−822.64; −148.82]

Yanqin Sun	6MWT	*p* < 0.05	95.18 (76.89)	53.4 (73.52)	41.78 [14.86; 68.70]

Peng Deng	LVEF	*p* < 0.05	4.59 (7.73)	3.69 (7.21)	0.9 [−2.38; 4.18]
	BNP	*p* < 0.05	−482.77 (312.8)	−147 (300.02)	−335.77 [−470.09; −201.45]
	6MWT	*p* < 0.05	75.63 (28.52)	40 (27.95)	35.63 [23.26; 48.00]

Jing Wang	HR	*p* < 0.01	−15 (7.55)	−12 (9.03)	−3 [−7.21; 1.21]
	CO	*p* < 0.01	1.6 (0.61)	1 (0.56)	0.6 [0.30; 0.90]
	LVEF	*p* < 0.01	17.5 (8.12)	8.6 (7.92)	8.9 [4.84; 12.96]

Yongjian Wen	BNP	*p* < 0.05	−224.25 (147.15)	−135.54 (234.49)	−88.71 [−155.00; −22.42]
	HR	*p* < 0.05	−4.11 (3.63)	−1.94 (3.41)	−2.17 [−7.21; 1.21]
	LVEF	*p* < 0.05	8.74 (10.02)	5.27 (10.18)	3.47 [0.05; 6.89]
	LVEDD	*p* < 0.05	−3.03 (8.02)	−2.15 (7.82)	−0.88 [−3.56; 1.80]

Xin li	HR	*p* < 0.05	−13.48 (8.2)	−6.93 (7.86)	−6.55 [−10.31; −2.79]
	NT-proBNP	*p* < 0.05	−1980.53 (243.87)	−1418.11 (211.46)	−562.42 [−669.36; −455.48]
	CO	*p* < 0.05	1.21 (0.37)	0.91 (0.41)	0.3 [0.12; 0.48]
	LVEF	*p* < 0.05	13.11 (6.7)	7.67 (6.96)	5.44 [2.24; 8.64]

Dongqun Lin	CO	*p* < 0.05	2.36 (0.68)	0.28 (0.63)	2.08 [1.75; 2.41]
	CI	*p* < 0.05	1.62 (0.44)	0.55 (0.55)	1.07 [0.82; 1.32]
	SV	*p* < 0.05	24.23 (4.6)	10.37 (4.52)	13.86 [11.59; 16.13]

Xiaofeng Zheng	NT-proBNP	*p* < 0.05	−1953.16 (1358.15)	−1162.14 (1226.07)	−791.02 [−1456.95; −125.09]

Weidong Huang	EF	*p* < 0.05	11.91 (5.06)	9 (3.53)	8.38 [5.09; 11.67]
	E/A	*p* < 0.01	0.23 (0.22)	0.01 (0.19)	0.22 [0.09; 0.35]

Leiqun Cheng	LVEDD	*p* < 0.05	−5.39 (4.94)	−1.76 (4.98)	−3.63 [−5.57; −1.69]
	LVEF	*p* < 0.05	11.77 (4.74)	6.01 (4.81)	5.76 [3.89; 7.63]
	E/A	*p* < 0.05	0.19 (0.17)	0.09 (0.17)	0.1 [0.03; 0.17]
	hsCRP	*p* < 0.05	−1.66 (1.65)	−0.84 (1.59)	−0.82 [−1.45; −0.19]

Wei Gao	BNP	*p* < 0.05	−379.27 (57.09)	−216.85 (51.26)	−162.42 [−186.20; −138.64]

Ziyong Li	HR	*p* > 0.05	−22.2 (18.62)	−18.6 (19.52)	−3.6 [−15.51; 8.31]
	MAP	*p* > 0.05	−14.2 (16.17)	−12.5 (16.29)	−1 [−9.95; 7.95]
	SI	*p* < 0.01	19.2 (6.21)	13.5 (6.21)	5.7 [1.83; 9.57]
	CI	*p* < 0.05	1.3 (0.38)	0.95 (0.39)	0.35 [0.11; 0.59]
	NT-proBNP	*p* < 0.05	−6162.7 (599.57)	−5666.3 (541.43)	−496.4 [−850.42; −142.38]

Chunbai Lai	LVEF	*p* < 0.05	20.8 (7.36)	10.9 (7.59)	9.9 [6.72; 13.08]

Lin Wang	LVEF	*p* < 0.05	23.6 (4.31)	12.4 (4.76)	11.2 [9.96; 12.44]
	CO	*p* < 0.05	1.8 (0.36)	0.6 (0.45)	1.2 [1.09; 1.31]

Yanli Rong	HR	*p* < 0.05	−15.1 (7.69)	−11.1 (9.34)	−4 [−7.75; −0.25]
	CO	*p* < 0.05	1.6 (0.44)	0.8 (0.69)	0.8 [0.55; 1.05]
	LVEF	*p* < 0.05	17.5 (8.28)	9.2 (7.97)	8.3 [4.74; 11.86]

Lisha Mai	6MWT	*p* < 0.05	119 (21)	73 (19.07)	46 [37.14; 54.86]

Qimei Zhang	EF	*p* < 0.01	10.11 (5.97)	4.73 (5.75)	5.38 [2.41; 8.35]
	HR	*p* < 0.01	−25.7 (7.69)	−19 (7.75)	−6.7 [−10.61; −2.79]
	LVESV	*p* < 0.01	−0.84 (88.2)	22.7 (23.13)	−23.54 [−56.17; 9.09]
	SV	*p* < 0.01	27.1 (9.51)	12.64 (10.46)	14.46 [9.40; 19.52]

Zhaojia Chen	BNP	*p* < 0.05	−201.89 (212.48)	432.67 (244.77)	−634.56 [−716.57; −552.55]
	HR	*p* < 0.05	−4.11 (2.68)	−1.93 (3.97)	−2.18 [−3.39; −0.97]

Jingjuan Yu	BNP	*p* < 0.05	−330.6 (336.92)	−261.6 (330.84)	−69 [−215.33; 77.33]

Dongmei Liu	LVESV	*p* < 0.05	20.69 (105.57)	−1.21 (21.32)	21.9 [−18.25; 62.05]
	LVEF	*p* < 0.05	12.38 (11.75)	2.13 (11.7)	10.25 [3.61; 16.89]

Yongchang Ma	LVEDD	*p* < 0.05	−13.33 (4.49)	−5.18 (4.7)	−8.15 [−10.16; −6.14]
	CO	*p* < 0.05	1.31 (1.83)	0.08 (2.03)	1.23 [0.38; 2.08]
	EF	*p* < 0.05	13.18 (3.36)	4.01 (3.91)	9.17 [7.57; 10.77]

Yanna Lei	EF	*p* < 0.05	9.3 (4.75)	1.4 (5.05)	7.9 [5.42; 10.38]

Xingliang Fan	EF	*p* < 0.05	7.83 (6.85)	5.08 (7.98)	2.75 [−0.85; 6.35]
	NT-proBNP	*p* > 0.05	−1461.11 (1567.87)	−1078.51 (1503.52)	−382.6 [−1123.62; 358.42]

Yong Zhi	EF	*p* < 0.05	14.1 (6.29)	8.1 (6.1)	6 [3.62; 8.38]
	BNP	*p* < 0.05	−4755 (70.42)	−4668.5 (67.18)	−86.5 [−112.95; −60.05]

Chunying Si	hsCRP	*p* < 0.05	−3.28 (1.93)	−1.67 (2.34)	−1.61 [−3.10; −0.12]
	6MWT	*p* < 0.05	63 (66.09)	29 (74.67)	34 [−14.86; 82.86]
	EF	*p* < 0.05	16.02 (3.13)	7.54 (3.54)	8.48 [6.16; 10.80]
	SV	*p* < 0.05	15.72 (3.57)	5.25 (3.29)	10.47 [8.09; 12.85]
	CO	*p* < 0.05	1.45 (0.35)	0.34 (0.33)	1 [0.87; 1.35]
	CI	*p* < 0.05	0.59 (0.3)	0.45 (0.31)	0.14 [−0.07; 0.35]

Haifeng Zhou	EF	*p* < 0.05	6.09 (3.85)	4.69 (3.73)	1.4 [0.01; 2.79]

Minyong Gan	6MWT	*p* < 0.05	211 (42.23)	156.6 (38.3)	54.4 [39.05; 69.75]

LVEF: left ventricular ejection fraction. LVEDD: left ventricular end-diastolic dimension. LVESV: left ventricular end-systolic volume. CO: cardiac output. SV: stroke volume. SI: stroke index CI: cardiac index. MAP: mean arterial pressure. BNP: brain natriuretic peptide. TNF-*α*: tumor necrosis factor-*α*. hsCRP: high-sensitivity C-reactive protein. 6MWT: six-minute walking test. Acu: acupuncture. Mox: moxibustion.

**Table 3:** Results of the subgroup analysis.

	Factor	Subgroup	No.	*I* ^2^ (%)	tau^2^	OR	[95% CI]	*p* value	Between-group *p* value
Efficacy	Total	—	13	0	0	2.61	[1.84; 3.72]	<0.0001^*∗*^	—
	Age	<60	3	0	0	3.23	[1.74; 5.99]	0.0002^*∗*^	0.45
		≥60	10	0	0	2.36	[1.53; 3.63]	<0.0001^*∗*^	
	NYHA	<III	3	0	0	2.36	[1.27; 4.40]	0.0068^*∗*^	0.62
		>III	1	—	—	1.85	[0.71; 4.79]	0.2	
		II∼IV	6	0	0	3.1	[1.76; 5.48]	<0.0001^*∗*^	

LVEF	Total	—	18	93	20.11	6.34	[4.11; 8.57]	<0.0001^*∗*^	—
	Disease	AHF	2	0	0	11.17	[9.95; 12.39]	<0.0001^*∗*^	<0.0001^*∗*^
		CHF	16	91	16.45	5.83	[3.67; 7.98]	<0.0001^*∗*^	
	NYHA	<III	2	0	0	−0.88	[−2.05; 0.30]	0.143	<0.0001^*∗*^
		＞III	2	84	36.58	5.11	[−4.01; 14.23]	0.272	
		II∼IV	9	37	1.22	6.55	[5.63; 7.48]	<0.0001^*∗*^	
	Intervention	Acupuncture	14	96	22	7.03	[4.46; 9.60]	<0.0001^*∗*^	0.76
		Moxibustion	4	76	10.24	5.78	[2.18; 9.38]	0.0016^*∗*^	
	Age	<60	5	98	37.29	6.35	[0.92; 11.79]	0.021^*∗*^	0.97
		≥60	13	69	5.31	6.45	[4.86; 8.05]	<0.0001^*∗*^	

HR	Total	—	9	24	0.61	−2.63	[−3.41; −1.86]	<0.0001^*∗*^	—
	Disease	AHF	2	0	0	−0.81	[−8.42; 6.80]	0.83	0.54
		CHF	7	40	0.98	−3.23	[−4.51; −1.93]	<0.0001^*∗*^	
	NYHA	<III	1	—	—	2	[−10.43; 14.43]	0.75	0.7
		＞III	2	0	0	−2.93	[−10.42; 4.55]	0.44	
		II∼IV	6	50	1.23	−3.3	[−4.68; −1.92]	<0.0001^*∗*^	
	Intervention	Acupuncture	6	8	0.14	−2.37	[−3.19; −1.54]	<0.0001^*∗*^	0.064
		Moxibustion	3	0	0	−4.62	[−6.87; −2.38]	<0.0001^*∗*^	
	Age	<60	2	0	0	−2.14	[−3.35; −0.93]	0.0005^*∗*^	0.11
		≥60	7	0	0	−3.87	[−5.61; −2.12]	<0.0001^*∗*^	

LVEDD	Total	—	3	90	11.31	−4.29	[−8.31; −0.27]	0.036^*∗*^	—
	Age	<60	1	—	—	−3.63	[−5.57; −1.69]	0.0003^*∗*^	0.036^*∗*^
		≥60	2	34	24.96	−4.57	[−11.69; 2.55]	0.2	

LVESV	Total	—	2	66	683.96	−2.39	[−46.81; 42.04]	0.91	—
	Disease	AHF	1	—	—	21.9	[−18.25; 62.05]	0.29	0.91
		CHF	1	—	—	−23.54	[−56.17; 9.09]	0.16	
	NYHA	＞III	1	—	—	21.9	[−18.25; 62.05]	0.29	0.91
		II∼IV	1	—	—	−23.54	[−56.17; 9.09]	0.16	

CO	Total	—	8	94	0.25	1.02	[0.65; 1.39]	<0.0001^*∗*^	—
	Disease	AHF	2	96	0.37	1.63	[0.76; 2.49]	<0.0001^*∗*^	0.073
		CHF	6	85	0.12	0.78	[0.46; 1.11]	<0.0001^*∗*^	
	NYHA	<III	—	—	—	—	—	—	0.62
		＞III	1	—	—	0.98	[0.42; 1.54]	0.0007^*∗*^	
		II∼IV	4	97	0.48	0.94	[0.24; 1.63]	0.0082^*∗*^	
	Intervention	Acupuncture	5	86	0.12	1.34	[0.98; 1.70]	<0.0001^*∗*^	0.0013^*∗*^
		Moxibustion	3	81	0.06	0.56	[0.24; 0.88]	0.0006^*∗*^	
	Age	<60	2	0	0	1.19	[1.08; 1.30]	<0.0001^*∗*^	0.47
		≥60	6	95	0.36	1	[0.50; 1.51]	0.0001^*∗*^	

CI	Total	—	5	88	0.12	0.51	[0.18; 0.84]	0.0026^*∗*^	—
	Disease	AHF	2	94	0.21	0.73	[0.08; 1.39]	0.0287^*∗*^	0.2411
		CHF	3	54	0.02	0.32	[0.08; 0.55]	0.0087^*∗*^	
	NYHA	<III	—	—	—	—	—	—	0.0648
		＞III	2	0	0	0.45	[0.25; 0.65]	<0.0001^*∗*^	
		II∼IV	2	94	0.24	0.71	[0.01; 1.41]	0.0488^*∗*^	
	Age	<60	1	—	—	0.62	[0.18; 1.06]	0.0055^*∗*^	0.6532
		≥60	4	91	0.14	0.49	[0.11; 0.87]	0.0126^*∗*^	

SI	Total	—	2	0	0	5.1	[2.65; 7.55]	<0.0001^*∗*^	—
	Disease	AHF	1	—	—	4.7	[1.53; 7.87]	0.0036^*∗*^	0.69
		CHF	1	—	—	5.7	[1.83; 9.57]	0.0039^*∗*^	
	NYHA	<III	—	—	—	—	—	—	0.69
		＞III	1	—	—	4.7	[1.53; 7.87]	0.0036^*∗*^	
		II∼IV	1	—	—	5.7	[1.83; 9.57]	0.0039^*∗*^	

SV	Total	—	4	37	1.64	12.41	[10.91; 13.92]	<0.0001^*∗*^	—
	Disease	AHF	1	—	—	13.86	[11.59; 16.13]	<0.0001^*∗*^	0.096
		CHF	3	1	0.04	11.31	[9.28; 13.33]	<0.0001^*∗*^	
	NYHA	<III	—	—	—	—	—	—	0.093
		＞III	1	—	—	11.93	[6.52; 17.34]	<0.0001^*∗*^	
		II∼IV	2	0	0	13.96	[11.89; 16.03]	<0.0001^*∗*^	
	Age	<60	1	—	—	11.93	[6.52; 17.34]	<0.0001^*∗*^	0.824
		≥60	3	58	3.01	12.61	[9.98; 15.23]	<0.0001^*∗*^	

NT-proBNP	Total	—	5	0	0	−553.05	[−649.14; −456.97]	<0.0001^*∗*^	—
	Disease	AHF	1	—	—	−791.02	[−1456.95; −125.09]	0.0199^*∗*^	0.4791
		CHF	4	0	0	−548	[−645.10; −450.90]	<0.0001^*∗*^	
	NYHA	<III	—	—	—	—	—	—	0.8491
		＞III	2	0	0	−561.32	[−873.91; −248.72]	0.0004	
		II∼IV	1	—	—	−562.42	[−669.36; −455.48]	<0.0001^*∗*^	
	Intervention	Acupuncture	3	0	0	−534.32	[−822.34; −246.30]	0.0003^*∗*^	0.8924
		Moxibustion	2	0	0	−555.4	[−657.33; −453.48]	<0.0001^*∗*^	

BNP	Total	—	7	96	18700	−227.99	[−337.30; −118.68]	<0.0001^*∗*^	—
	NYHA	<III	2	0	0	−163.21	[−186.85; −139.58]	<0.0001^*∗*^	0.0392^*∗*^
		＞III	1	—	—	−335.77	[−470.09; −201.45]	<0.0001^*∗*^	
		II∼IV	3	99	63828.54	−267.88	[−556.04; 20.28]	<0.0001^*∗*^	
	Intervention	Acupuncture	5	98	18881.11	−209.08	[−334.48; −83.69]	<0.0001^*∗*^	0.2607
		Moxibustion	2	0	0	−306.45	[−420.71; −192.18]	<0.0001^*∗*^	
	Age	<60	2	99	149218.3	−359.1	[−896.18; 177.98]	0.19	0.4757
		≥60	5	69	3756.15	−161.94	[−233.60; −90.27]	<0.0001^*∗*^	

CRP	Total	—	3	84	0.84	−1.61	[−2.78; −0.45]	0.0068^*∗*^	—
	NYHA	<III	1	—	—	−2.4	[−3.01; −1.80]	<0.0001^*∗*^	0.002^*∗*^
		＞III	—	—	—	—	—	—	
		II∼IV	1	—	—	−1.61	[−3.10; −0.12]	0.0113^*∗*^	
	Age	<60	1	—	—	−0.82	[−1.45; −0.19]	0.0113^*∗*^	0.0007^*∗*^
		≥60	2	0	0	−2.29	[−2.85; −1.72]	<0.0001^*∗*^	

MAP	Total	—	4	0	0	−1.57	[−6.02; 2.88]	0.4896	—
	Disease	AHF	3	0	0	−1.54	[−6.50; 3.42]	0.5432	0.9776
		CHF	1	—	—	−1.7	[−11.81; 8.41]	0.7418	
	NYHA	<III	2	0	0	−2.03	[−8.27; 4.21]	0.5243	0.8376
		≥III	2	0	0	−1.1	[−7.45; 5.26]	0.7357	
		II∼IV	—	—	—	—	—	—	
	Age	<60	2	0	0	−2.03	[−8.27; 4.21]	0.5243	0.8376
		≥60	2	0	0	−1.1	[−7.45; 5.26]	0.7357	

6MWT	Total	—	7	0	0	43.6	[37.43; 49.77]	<0.0001^*∗*^	—
	NYHA	<III	3	0	0	34.83	[14.07; 55.60]	0.001^*∗*^	0.2901
		＞III	1	—	—	35.63	[23.26; 48.00]	<0.0001^*∗*^	
		II∼IV	2	0	0	48.1	[40.43; 55.77]	<0.0001^*∗*^	
	Intervention	Acupuncture	3	0	0	52.52	[37.94; 67.11]	<0.0001^*∗*^	0.1857
		Moxibustion	4	0	0	41.65	[34.84; 48.47]	<0.0001^*∗*^	
	Age	<60	1	—	—	41.78	[14.86; 68.70]	0.0023^*∗*^	0.8997
		≥60	6	6	4.87	43.7	[37.36; 50.04]	<0.0001^*∗*^	

Values could not be calculated due to an insufficient number of studies; ^*∗*^*p* > 0.05 was considered statistically significant; *p* value: *p* value between the study group and control group; between-group *p* value: *p* value between the subgroups.

**Table 4:** Metaregression.

Factor	No.	Duration			Times			Age		
		Estimate	SE	*p*	Estimate	SE	*p*	Estimate	SE	*p*
HR	10	−0.21	0.08	0.01^*∗*^	0.02	0.05	0.74	−0.08	0.05	0.11
LVEF	19	−0.16	0.11	0.13	−0.05	0.05	0.38	0.07	0.12	0.57
CO	8	−0.02	0.04	0.52	−0.01	0.01	0.32	−0.02	0.01	0.23
CI	5	0.00	0.04	0.95	−0.01	0.01	0.22	−0.01	0.02	0.44
NT-proBNP	5	−0.21	5.06	0.97	−1.28	5.38	0.81	−12.92	17.72	0.47
BNP	7	1.94	8.63	0.82	−8.21	2.61	0.00^*∗*^	11.12	11.45	0.33
6MWT	7	−0.01	0.07	0.84	−0.22	0.17	0.19	−0.53	0.70	0.45
Efficacy of acupuncture	18	0.00	0.04	0.99	0.00	0.02	0.87	−0.01	0.01	0.67
Efficacy of moxibustion	10	0.00	0.00	0.58	−0.01	0.01	0.53	−0.01	0.03	0.84
Efficacy of A&M	28	0.00	0.00	0.66	−0.01	0.01	0.54	0.00	0.01	0.68

^*∗*^ *p* < 0.05 was considered statistically significant.

## Supplementary Materials

Supplementary Materials Supplementary Figures S1 and S2 show the risk of bias. Figures S3–S9 show the forest plot of acupuncture and moxibustion effect on the following indicators: odds ratio, six-minute walking test, brain natriuretic peptide, left ventricular ejection fraction, cardiac output, and heart rate. Figures S10 and S11 show metaregression of heart rate and treatment duration, and metaregression of brain natriuretic peptide and treatment times. Figures S12 and S17 show the funnel plot of odds ratio, left ventricular ejection fraction, cardiac output, heart rate, six-minute walking test, and brain natriuretic peptide.
