# Supplementary material for: The Effect of Acupuncture and Moxibustion on Heart Function in Heart Failure Patients: A Systematic Review and Meta-Analysis
Source: Evid Based Complement Alternat Med. 2019 Oct 20;2019:6074967. doi: 10.1155/2019/6074967 (PMC6854931; doi:10.1155/2019/6074967)
Supplement: Supplementary Materials — Supplementary Figures S1 and S2 show the risk of bias. Figures S3–S9 show the forest plot of acupuncture and moxibustion effect on the following indicators: odds ratio, six-minute walking test, brain natriuretic peptide, left ventricular ejection fraction, cardiac output, and heart rate. Figures S10 and S11 show metaregression of heart rate and treatment duration, and metaregression of brain natriuretic peptide and treatment times. Figures S12 and S17 show the funnel plot of odds ratio, left ventricular ejection fraction, cardiac output, heart rate, six-minute walking test, and brain natriuretic peptide. [file 6074967.f1.docx]

**Figure S1 | Risk of bias summary**


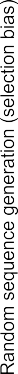

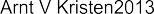

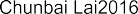

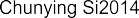

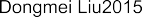

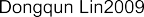

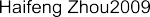

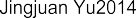

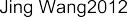

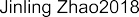

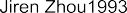

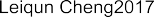

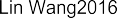

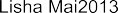

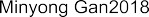

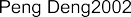

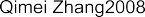

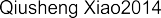

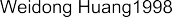

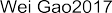

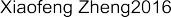

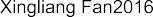

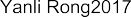

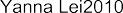

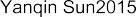

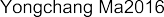

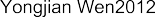

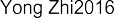

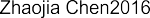

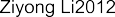

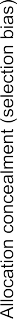

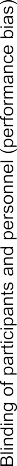

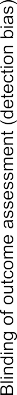

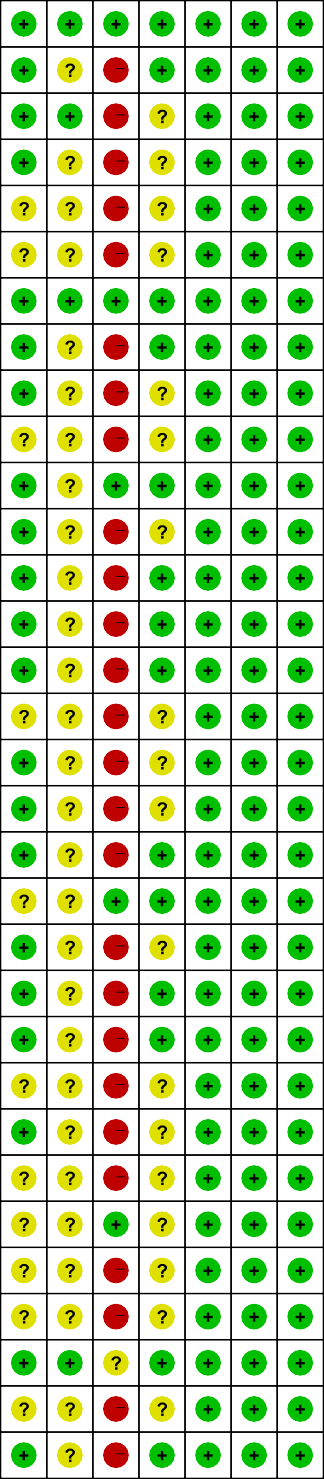

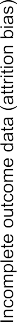

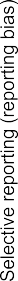

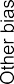


**Figure S2 | Risk of bias summary**


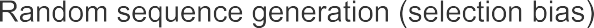

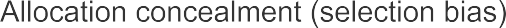

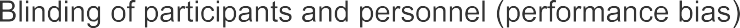

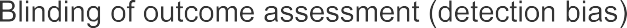

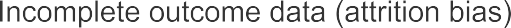

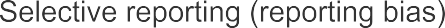

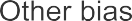

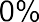

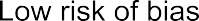

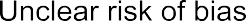

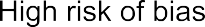


**Figure S3 |Forest plot of Odds Ratio**

**
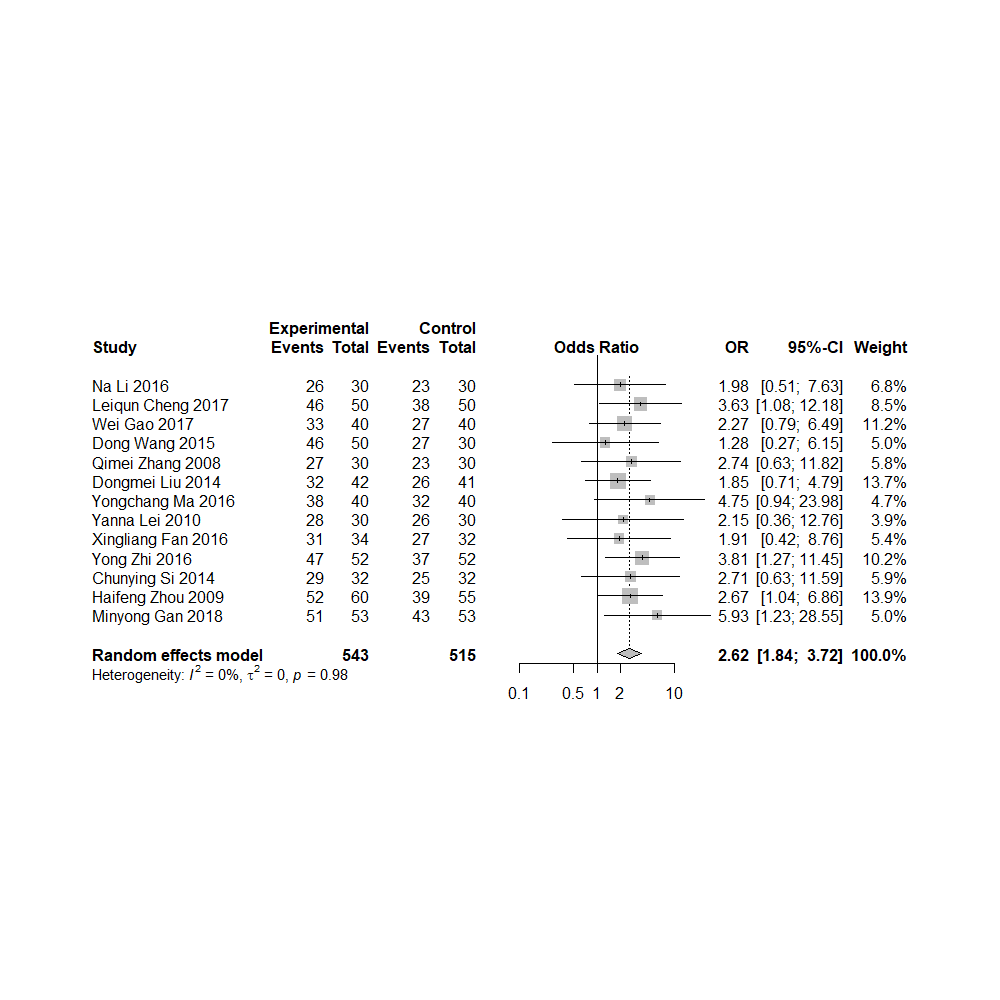
**

**Figure S4 |Forest plot of** **Six-Minutes Walking Test**

**
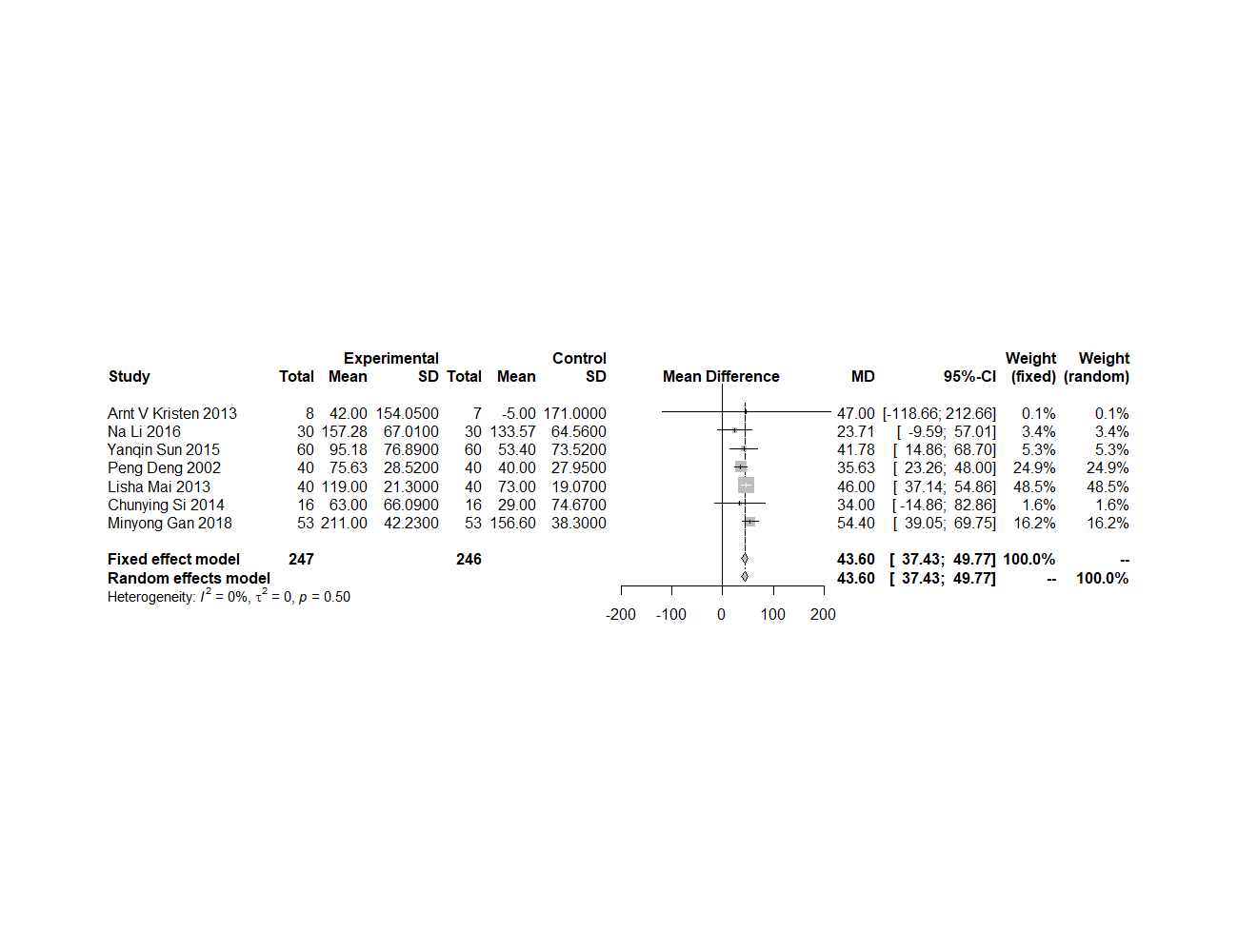
Figure S5 |Forest**

**plot of Brain Natriuretic Eptide**

**
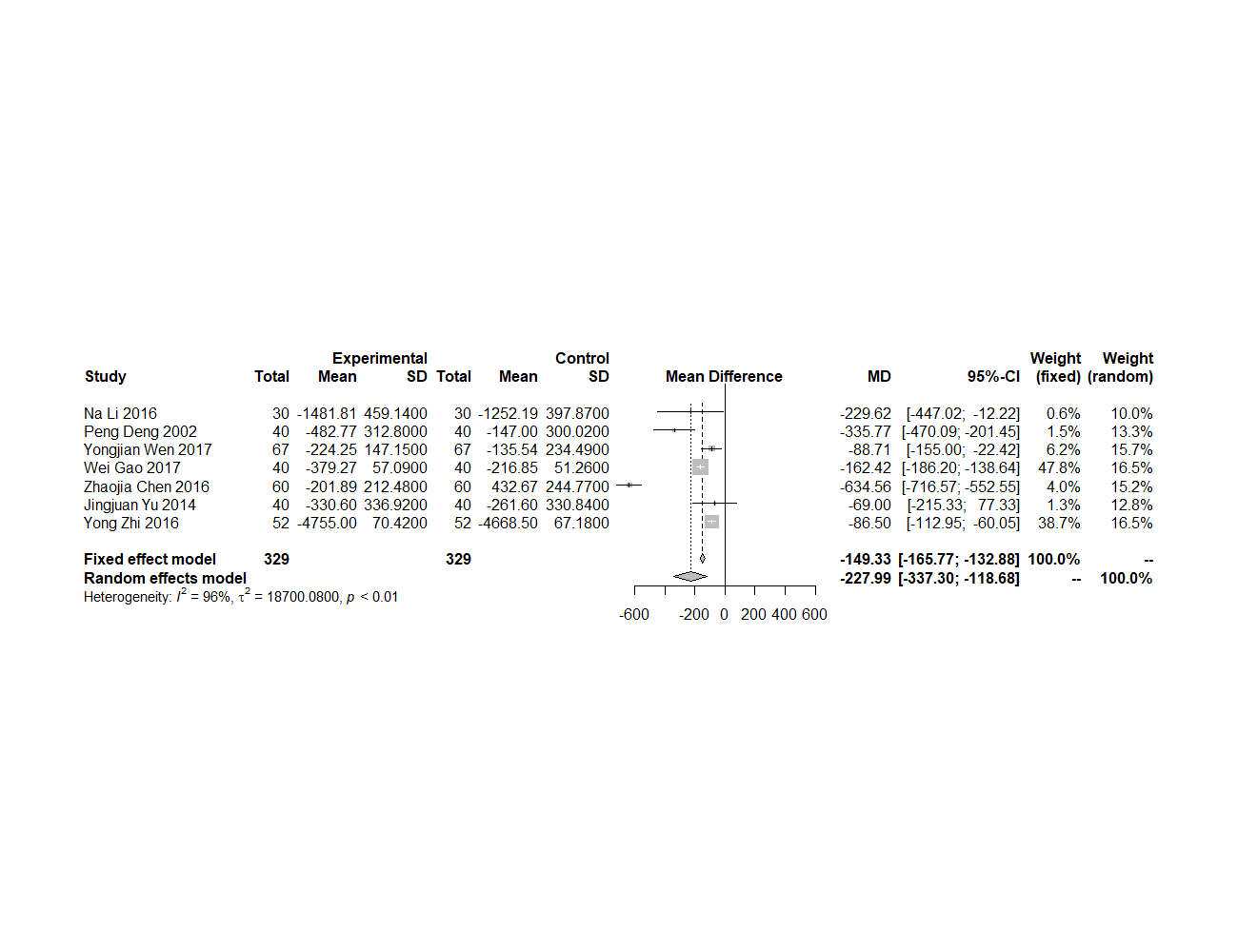
**

**Figure S6 |Forest plot of Left Ventricular Ejection Fraction**

**
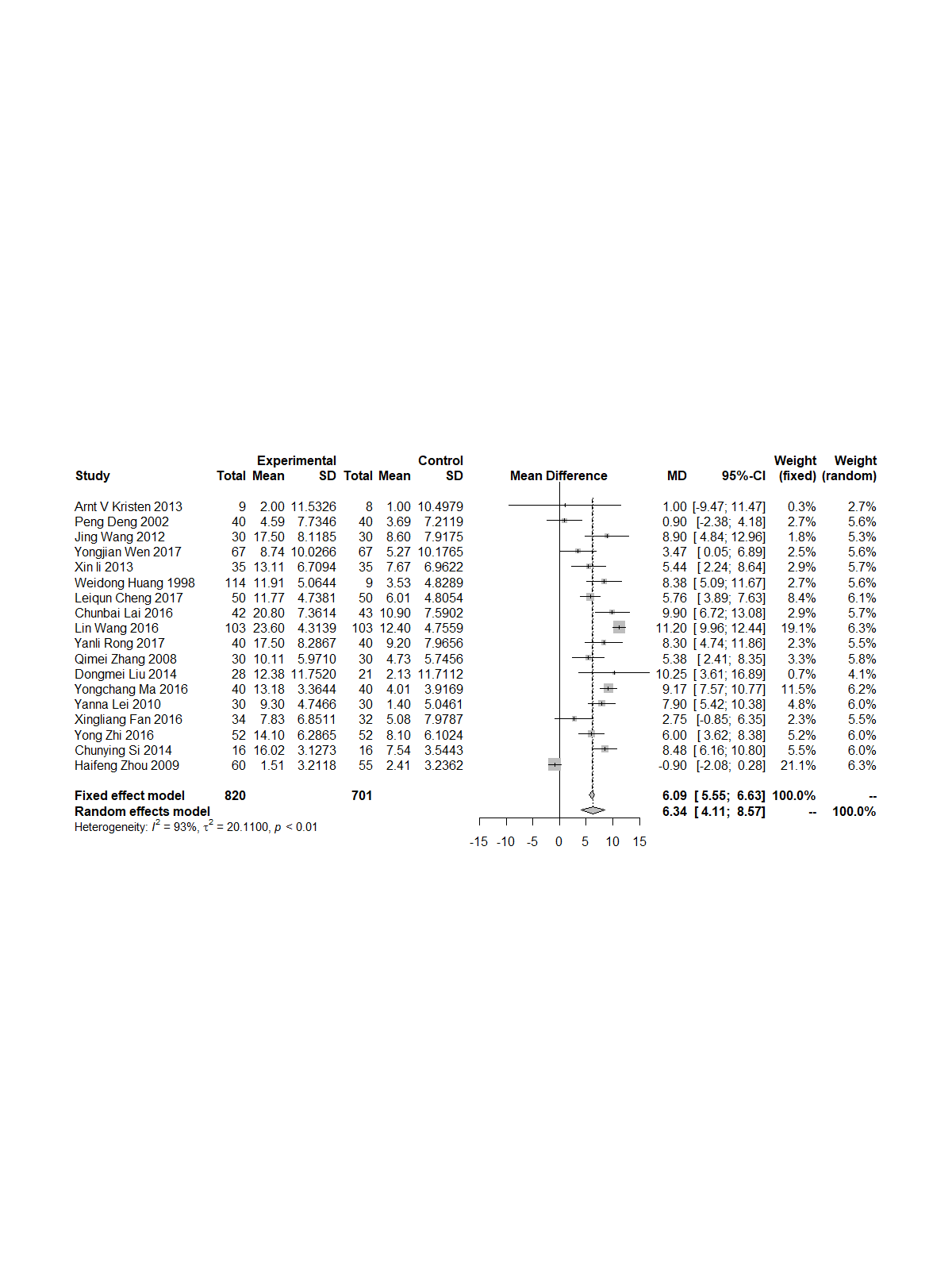
**

**Figure S7 |Forest plot of Left Ventricular Ejection Fraction of studies with ethical supervision**

**
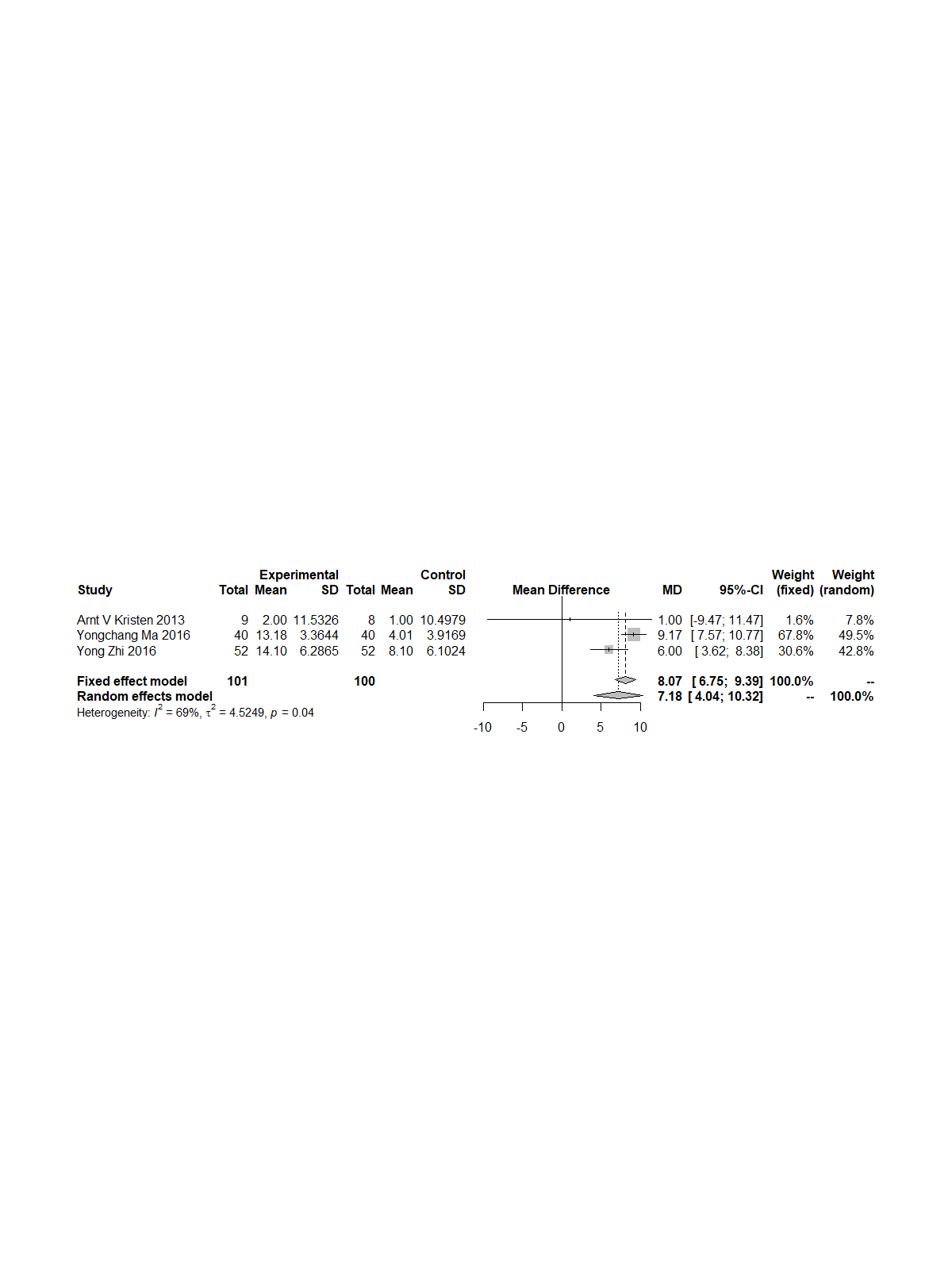
**

**Figure S8 |Forest plot of [Cardiac](D:/2345%E4%B8%8B%E8%BD%BD/Dict/7.5.2.0/resultui/dict/?keyword=cardiac)[Output](D:/2345%E4%B8%8B%E8%BD%BD/Dict/7.5.2.0/resultui/dict/?keyword=output)**

**
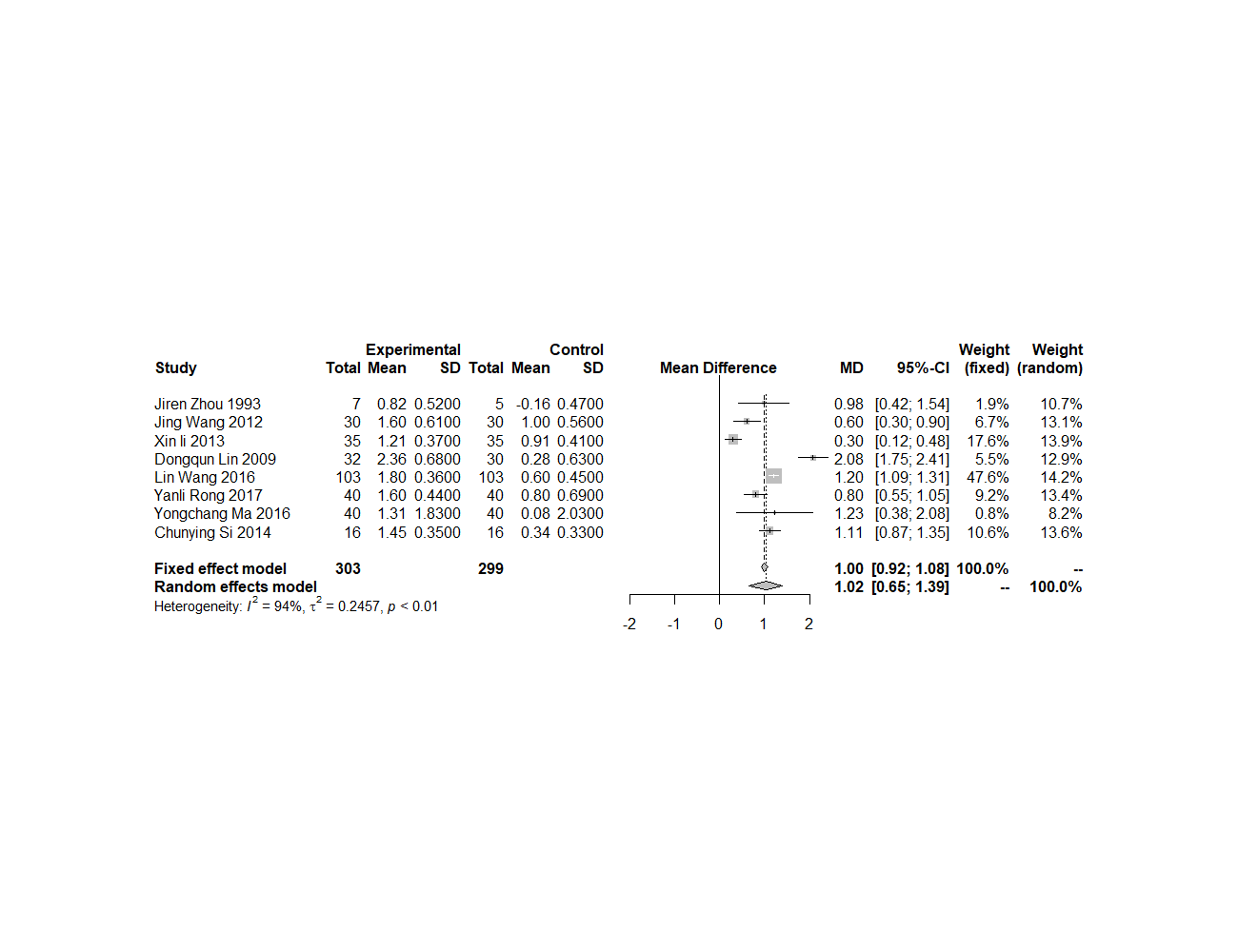
**

**Figure S9 |Forest plot of Heart Rate**

**
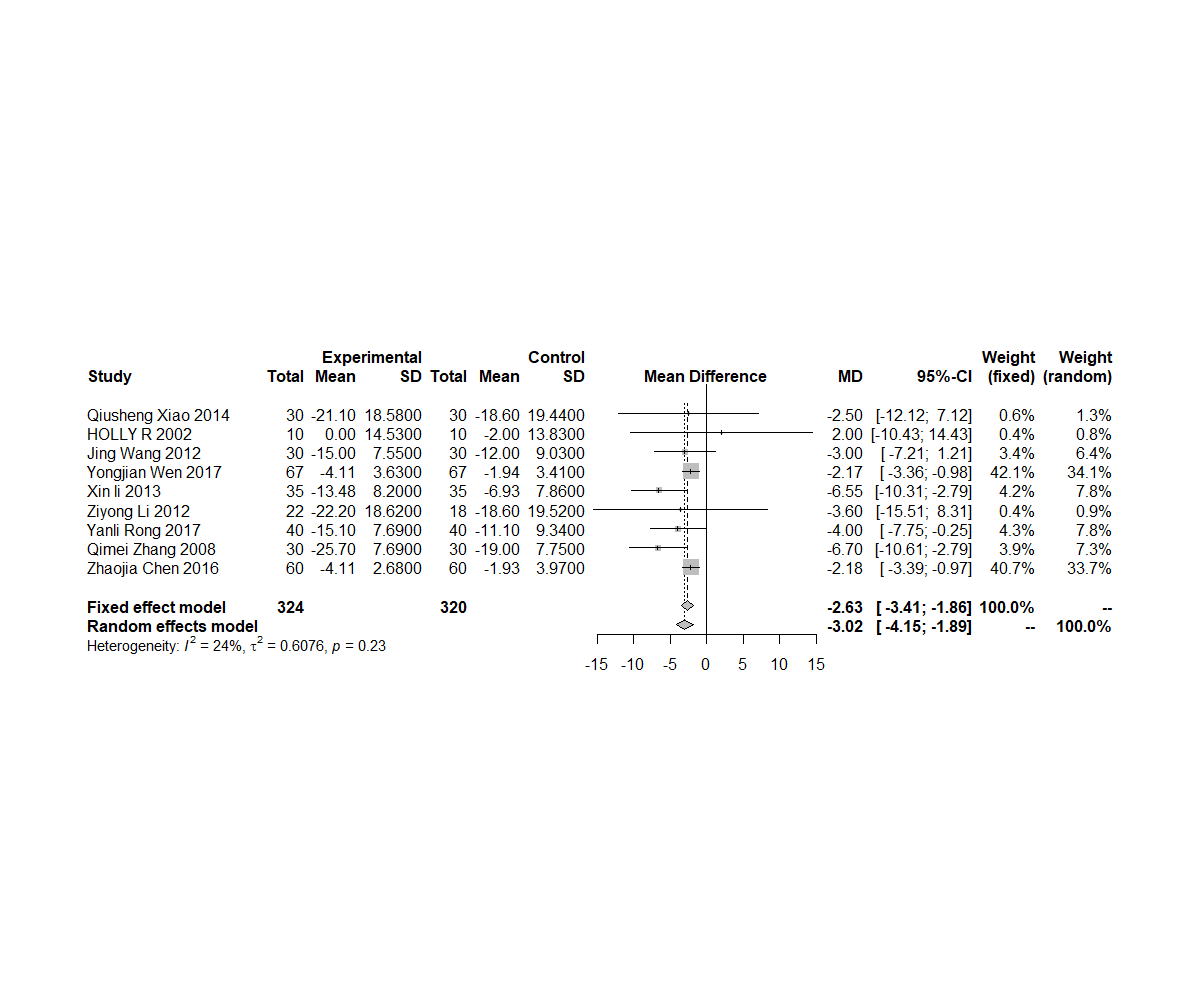
**

**Figure S10 | Meta-regression of Heart Rate and treatment duration**

**
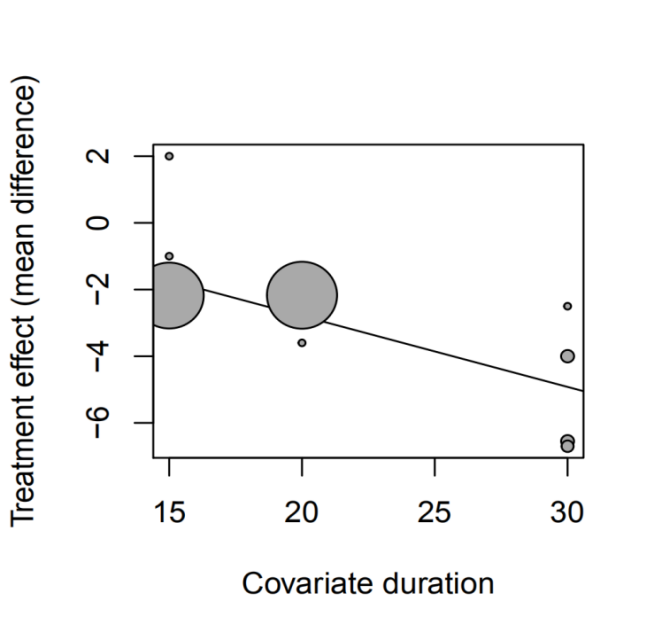
**

**Figure S11 | Meta-regression of Brain Natriuretic Eptide and treatment times**

**
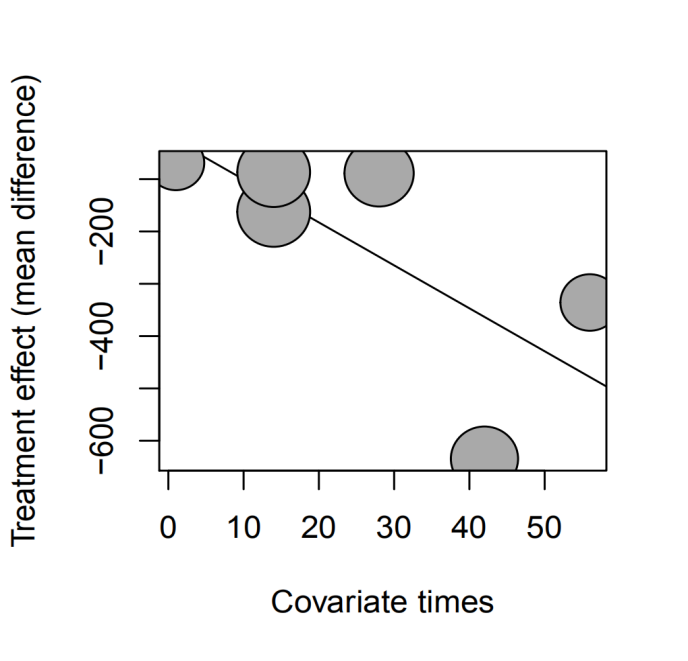
**

**Figure S12 | Funnel plot of Odds Ratio**

**
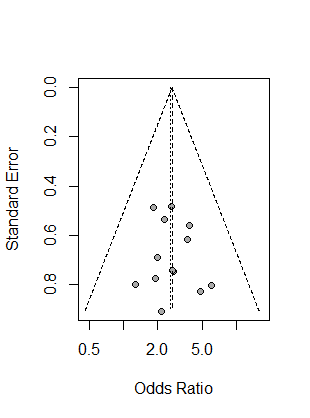
**

**Figure S13 | Funnel plot of Left Ventricular Ejection Fraction**

**
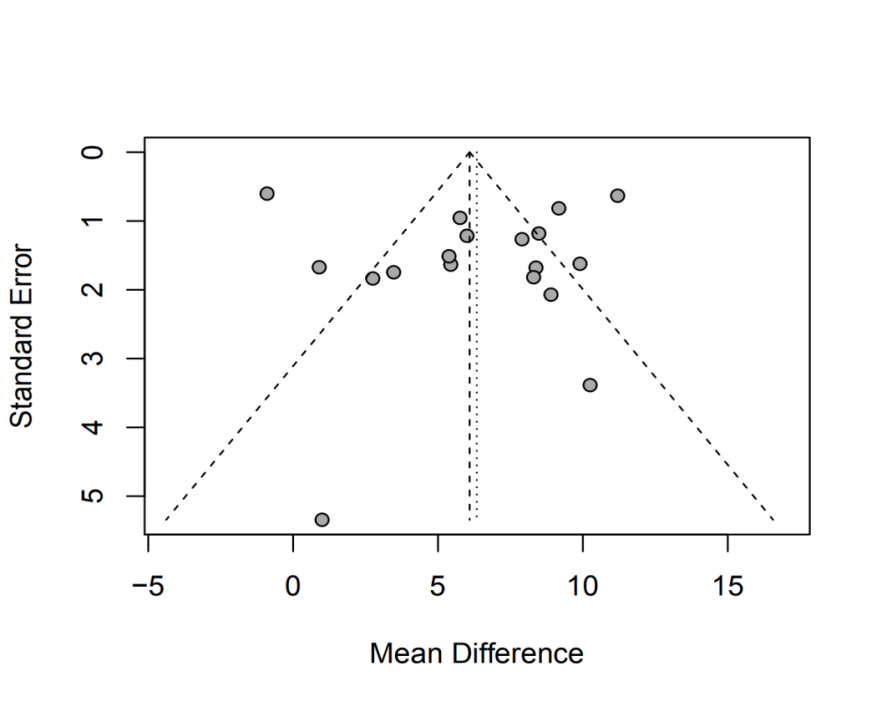
**

**Figure S14 | Funnel plot of [Cardiac](D:/2345%E4%B8%8B%E8%BD%BD/Dict/7.5.2.0/resultui/dict/?keyword=cardiac)[Output](D:/2345%E4%B8%8B%E8%BD%BD/Dict/7.5.2.0/resultui/dict/?keyword=output)**

**
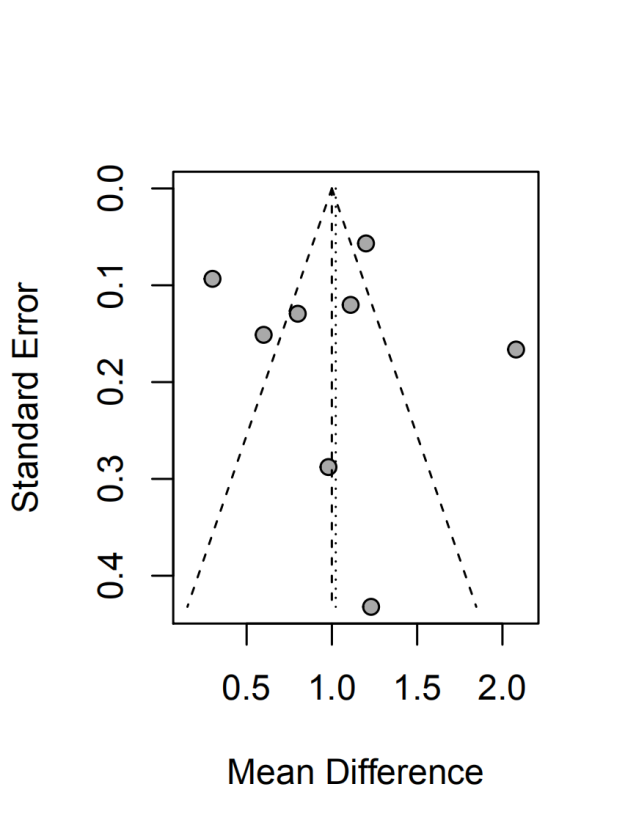
**

**Figure S15 | Funnel plot of Heart Rate**

**
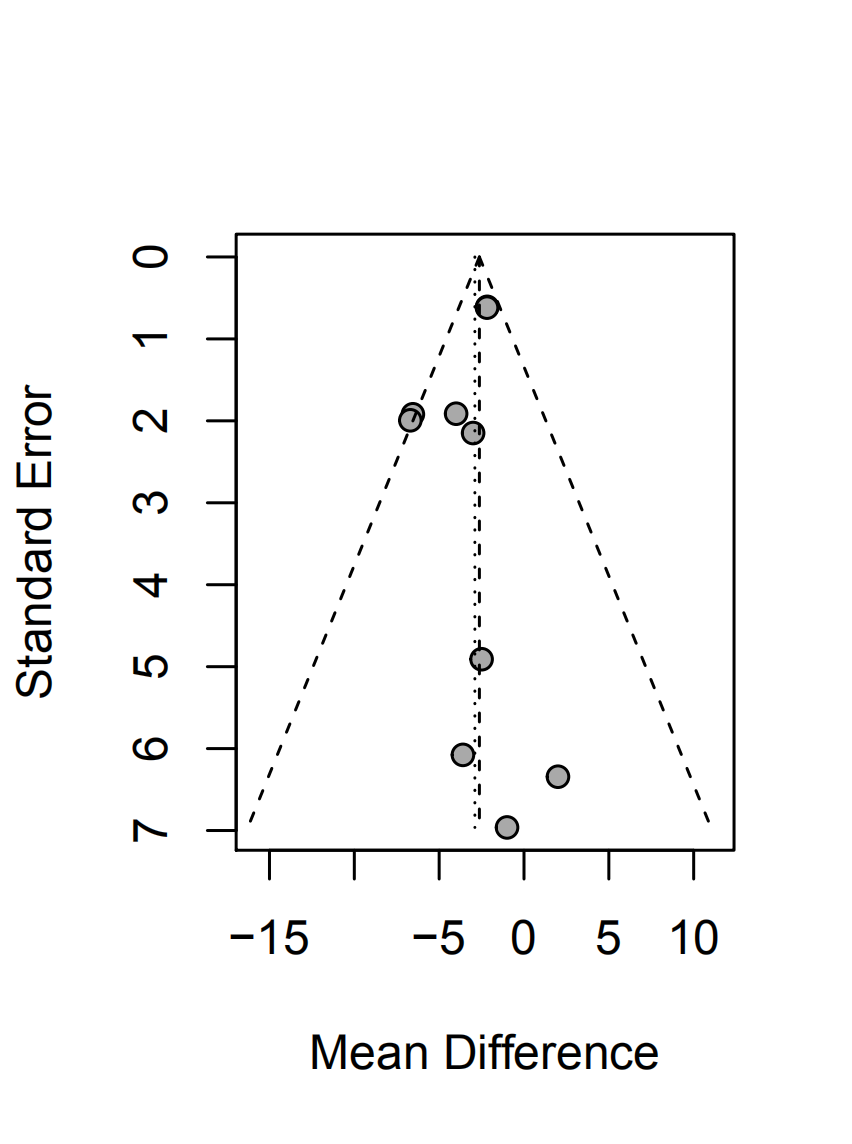
**

**Figure S16 | Funnel plot of Six-Minutes Walking Test**

**
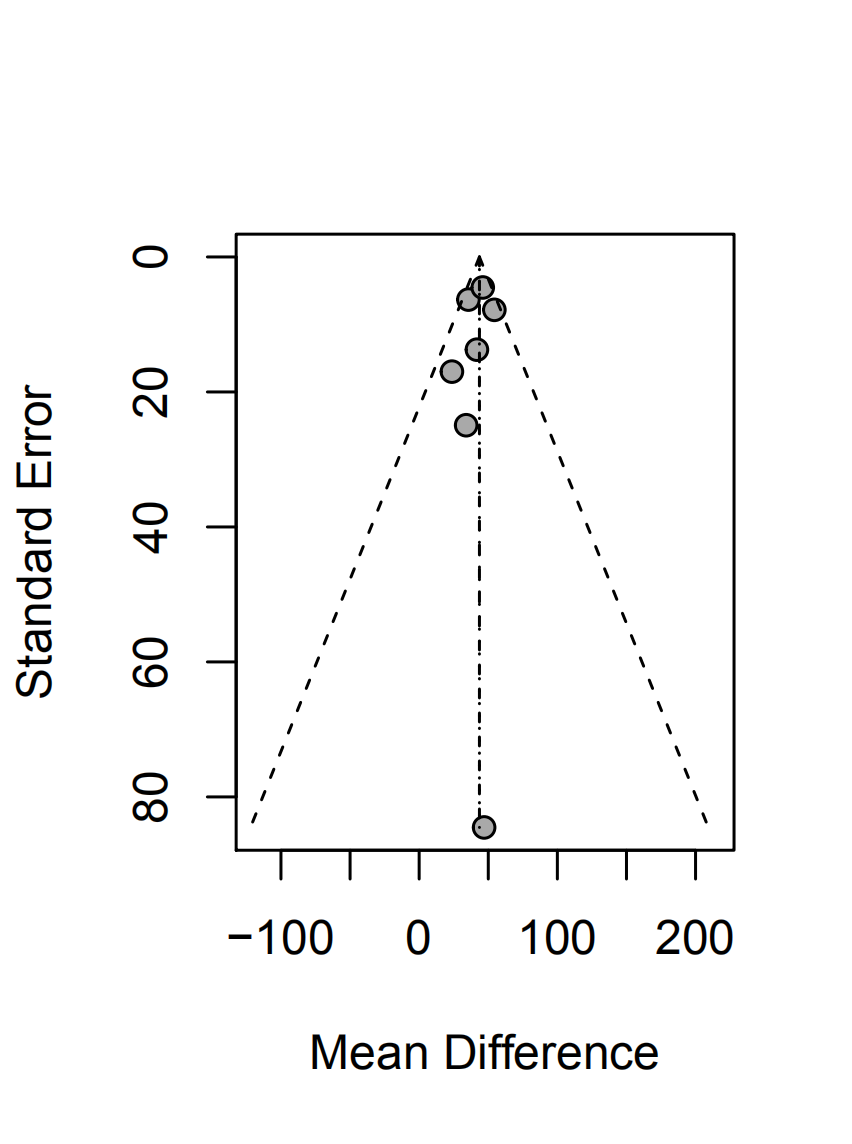
**

**Figure S17 | Funnel plot of Brain Natriuretic Eptide**

**
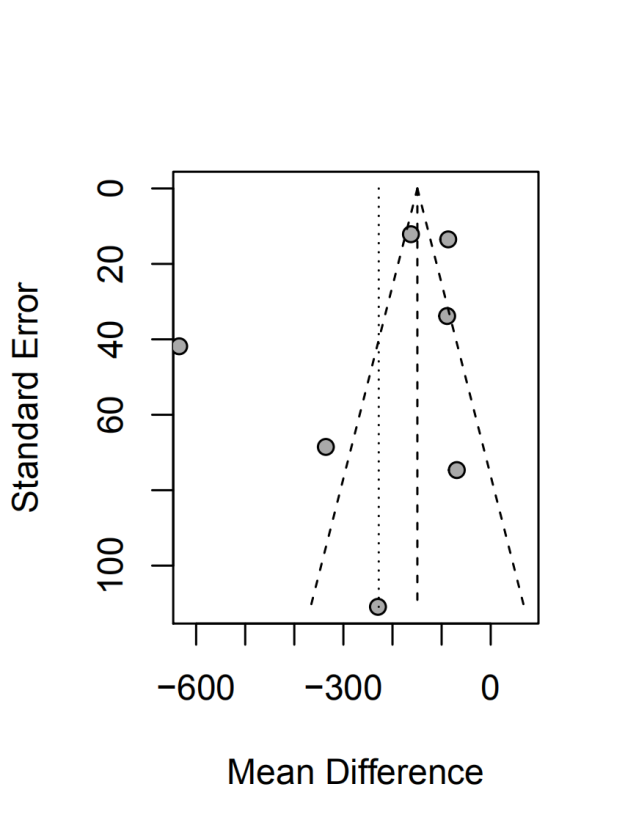
**
